# Evaluation of Nasal Obstruction Following Septoturbinoplasty Using the VAS and NOSE Scale

**DOI:** 10.22038/IJORL.2023.75824.3541

**Published:** 2024-01

**Authors:** Esteban Merino-Galvez, Javier Gómez-Hervás

**Affiliations:** 1 * Catholic University of Saint Anthony (Spain)*; 2 *Department of Ear, Nose and Throat. Hospital Rafael Méndez, Lorca, Spain.*

**Keywords:** Airway obstruction, Nasal obstruction, Nasal septum, Visual analogue scale

## Abstract

**Introduction:**

Patient satisfaction with septoturbinoplasty was measured using the subjective visual analogue scale (VAS) and Nasal Obstruction Symptom Evaluation (NOSE) scale. In addition, those factors that impacted satisfaction were confirmed.

**Materials and Methods:**

We conducted an observational study of patients who underwent septoturbinoplasty. Age, sex, smoking habit, duration of improvement, postoperative complications, type of packing and surgeon were analysed. The results were compared using the VAS and NOSE scale.

**Results:**

The improvement experienced with surgery corresponded to 69.80±26.97 points on the VAS 42.65±22.9 points (p <0.01) on the NOSE scale. A strong, direct correlation between the two scales (r = 0.79; p <0.01) was achieved. Surgeon, presence of complications, smoking habit and type of packing were not associated with the improvement experienced on the VAS or NOSE scale. Patients under 30 years of age and patients with permanent improvement achieved higher levels of satisfaction on both scales (p <0.01). Women showed a stronger tendency to perceive their improvement as temporary (p <0.01).

**Conclusions:**

Patients who underwent septoturbinoplasty experienced a subjectively measured improvement in nasal obstruction. The VAS and the NOSE scale were strongly correlated with one another. Sex, age and duration of improvement (temporary versus permanent) impacted patient perception; surgeon, smoking habit and type of packing did not.

## Introduction

Nasal obstruction is a highly prevalent disease responsible for a decrease in quality of life. Abnormal septal morphology, associated with inferior turbinate hypertrophy, is among the most common causes (1). Surgical treatment is the method of choice for managing this problem; the most commonly performed surgical procedure is septoplasty with turbinoplasty (2). There is evidence that nasal septum surgery is associated with better nasal airflow and resistance (3). It has also been reported that surgical correction is correlated with a subjective sense of improvement; (4) however, this is not always the case. Indeed, many studies have found that, despite improvement that could be measured with postoperative testing, patients' satisfaction with nasal breathing and their supposed improvement in quality of life fell short of expectations (5-6).

Reported factors that could alter patient-perceived outcomes include surgeon experience, suitable patient screening, technique used and type of packing. However, there are other factors beyond rhinological surgery itself, such as the results of non-validated instruments for measuring quality of life, proportions of eosinophils and even patients' baseline stress levels (7-9).

 Given all this, evaluation of outcomes in nasal obstruction surgery can be said to represent a major challenge. Validated instruments of measurement must be used to achieve a high level of evidence-based medicine. At present, there is no objective gold-standard test for evaluating surgical outcomes; consequently, subjective scales are used in clinical practice (10). The most commonly used among them have been the visual analogue scale (VAS) and Nasal Obstruction Symptom Evaluation (NOSE) scale. Both scales are considered key to measuring quality of life in these patients; however, uncertainties remain as to the best way to use these instruments (10). 

The primary objective of this study was to evaluate the repercussions of surgical treatment of nasal obstruction due to septal dysmorphia for patient satisfaction using the VAS and NOSE scale. The degree of correlation between perceived improvement reflected by the VAS and the same reflected by the NOSE scale was also analysed. In addition, the question of whether other factors such as sex, age, type of technique used and surgeon experience significantly influence the end result was answered.

## Materials and Methods

*Study design:*

An observational, retrospective study was conducted. It was approved by the ethics committee at Hospital Universitario Rafael Méndez [Rafael Méndez University Hospital].

*Patients: *Patients over 18 years of age who underwent septoplasty plus turbinoplasty due to septal deviation and turbinate hypertrophy at Hospital Universitario Rafael Méndez between 2016 and 2019, inclusive, were enrolled. Patients with nasal obstruction of an aetiology other than septal dysmorphia with associated turbinate hypertrophy were excluded. Patients with a diagnosis of acute or chronic sinusitis, nasal tumour, prior radiotherapy to the head or neck, septoplasty with rhinoplasty, craniofacial malformations, nasal trauma, or sarcoidosis or other granulomatous diseases were also excluded. 

Diagnoses were made and surgeries were performed by a single team of seven otorhinolaryngologists (37-62 years of age). In all cases, the surgical technique used was the classic Cottle's technique.

*Study variables: *A total of 98 patients were enrolled. Both sociodemographic variables (age, sex and year of operation) and clinical variables (type of packing, months elapsed between operation and evaluation, smoking habit, postoperative complications, and surgeon) were collected from the patients' medical records. In the first quarter of 2020, a blinded investigator evaluated the patients by way of an interview. 

He used the NOSE scale validated in Spanish by Larrosa et al., with a range from 0 to 100 (least obstruction to most). He calculated both the postoperative and (retrospectively) the preoperative score. He also estimated the improvement experienced in nasal obstruction with surgery using the VAS, with a range from 0 to 100 (least improvement experienced to most). 

He asked the patients about the nature of that improvement (whether it was stable or temporary and, if temporary, how long their improvement lasted in terms of number of months), days to recovery of their normal lives and, finally, how they felt at the time of the interview about their nasal obstruction - whether it was better than, worse than or the same as it was before surgery.

*Statistical analysis: *First, frequency analysis was performed. Next, qualitative variables were compared using the chi-squared test and means were compared using Student's t test and analysis of variance (ANOVA). Finally, a bivariate correlation study was performed on some variables. All results were considered significant for an alpha (α) error of <0.05. Statistical analysis was performed using the program SPSS version 21 from IBM.

## Results

A total of 112 patients met the inclusion criteria; 14 patients were lost due to lack of data. Of them, 88.8% were males. Their mean age was 37±12 with a range of 16-71 years; 34.7% of them were smokers. The type of nasal packing used was Merocel® in 73.5% of patients and gauze in 26.5%. With regard to postoperative complications, 3.5% visited the emergency department due to pain, 3.1% had epistaxis and 2.4% presented nasal adhesions.

 No septal perforations were seen. The mean number of days that elapsed before patients resumed their normal occupational and social lives was 30.5±9.27 days.While 84.7% of patients stated that they felt better than they did before surgery with regard to their nasal obstruction, 15.3% did not notice an improvement. Among those who reported an improvement, 71.4% experienced a permanent improvement, whereas 28.6% experienced just a temporary improvement with a mean duration of 4.24 months. 

**Fig 1:**
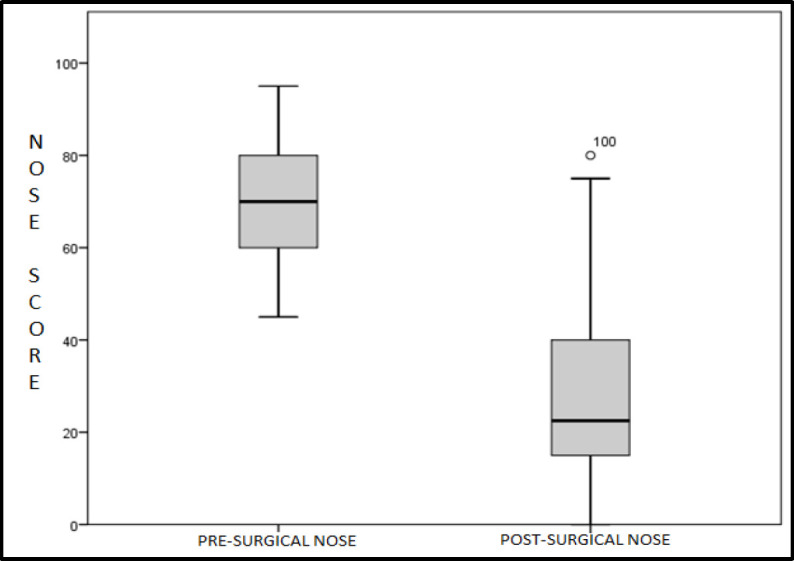
Comparison of pre- and post- surgical NOSE values

When the data were disaggregated by sex, it was found that 54.5% of the women who improved did so only temporarily; this figure was 28.6% in the group of men (p = 0.04). Regarding the scales used, the preoperative NOSE scale (70.46±12.20) was higher than the postoperative NOSE scale (27.81±19.59) (p <0.01) (Figure 1), with surgery achieving an improvement on the NOSE scale of 42.65±22.9 points.

**Fig 2:**
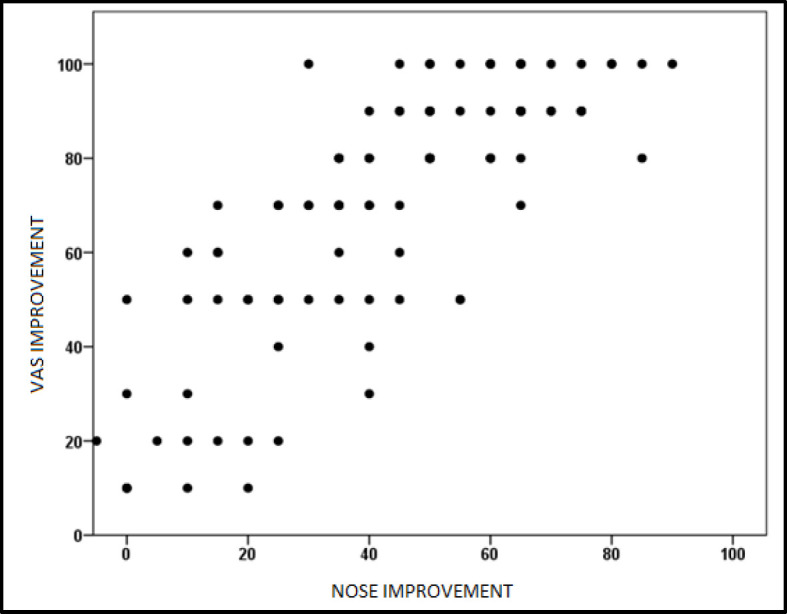
Correlation between the improvement perceived by the patient after surgery with the VAS and NOSE scales

The assessment of improvement in nasal obstruction with surgical treatment on the VAS was 69.80±26.97 points. The correlation between the percentage of improvement according to the NOSE scale and the VAS was direct and strong (r = 0.79, p <0.01) (Figure 2).

**Fig 3:**
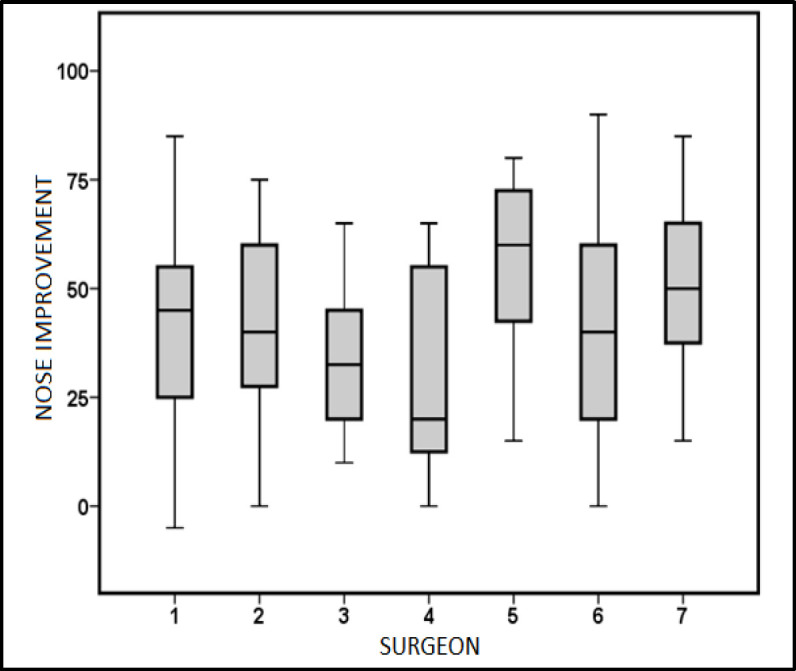
Comparison of the improvement perceived by the patient on the NOSE scale according to the surgeon who performed the intervention

When the results were compared by surgeon, no significant differences were found in perception of nasal obstruction measured with the NOSE scale (p = 0.23) or with the VAS (p = 0.16) in ANOVA (Figure 3). 

Furthermore, no differences were found depending on sex (p = 0.21 and p = 0.35, respectively), type of nasal packing (p = 0.43 and p = 0.16, respectively) or presence of postoperative complications (p = 0.34 and p = 0.12, respectively). However, significant differences were found in duration of improvement. Those with permanent improvement had better scores on the VAS (p <0.01) and NOSE scale (p <0.01). 

In line with this, those who reported no improvement in nasal obstruction after undergoing surgery had worse scores both on the VAS (p <0.01) and on the NOSE scale (p <0.01). In addition, a significant difference was seen between age and improvement achieved on the VAS and NOSE scale: age under 30 years was associated with greater perceived improvement (p <0.01). Table 1. 

**Table 1:** Comparison of patient-perceived improvement in nasal obstruction with surgery, using the NOSE scale (pre-surgical NOSE - post-surgical NOSE) and VAS. Both scales from 0 to 100 points; 0 points no improvement and 100 points complete improvement

	**Variable**	**Variable values**	**Points of improvement in nasal obstruction on both scales**				
			** NOSE escale**	**P value**	**EVA escale**	**P value**	
	Gender	Male	43,68 ± 21,70	p = 0,21	70,79 ± 26,72	p = 0,35	
		Female	34,55 ± 30,94		62,73 ± 30,03		
	Surgeon	Surgeon 1	41,38 ± 24,48	p = 0,23	70,00 ± 27,22	p = 0,16	
		Surgeon 2	40,66 ± 23,74		71,33 ± 24,74		
		Surgeon 3	34,00 ± 17,28		71,00 ± 25,58		
		Surgeon 4	31,42 ± 26,25		50,00 ± 38,73		
		Surgeon 5	55,62 ± 23,36		80,00 ± 23,29		
		Surgeon 6	40,00 ± 27,44		57,50 ± 31,30		
		Surgeon 7	49,16 ± 17,04		78,75 ± 18,72		
	Type of improvement	Temporary	19,46 ± 15,11	p<0,01	43,21 ± 24,80	p<0,01	
		Permanent	51,93 ± 18,51		80,43 ± 19,51		
	Clogging	Merocel®	43,75 ± 22,15	p = 0,43	72,08 ± 25,94	p = 0,16	
		Gauze	39,62 ± 25,09		63,46 ± 29,25		
	Age	Over 30	30,00 ± 26,03	p<0,01	57,89 ± 30,107	p<0,01	
		Under 30	47,01 ± 21,86		74,93 ± 25,724		
	Post-surgical complications	No	43,86 ± 22,65	p=0,34	70,87 ± 26,50	p = 0,12	
		Yes	24,17 ± 20,10		31,41 ± 26,97		
	Current status	Imprrovement	47,53 ± 20,32	p<0,01	77,47 ± 20,47	p<0,01	
		No improvement	15,67 ± 17,20		27,33 ± 17,51		
	Smoker	Yes	44,12 ± 24,323	p = 0,64	72,35 ± 28,716	p = 0,49	
		No	41,88 ± 22,281		68,44 ± 26,139		

## Discussion

Nasal obstruction is a condition directly related to patient-perceived quality of life (9). Evaluation of outcomes of septal surgery represents a challenge for the clinician in the absence of tools capable of predicting surgical outcomes (10). There are various instruments for calculating nasal airflow, such as active anterior rhinomanometry and peak inspiratory flow rate meters. These systems often do not demonstrate good correlation with subjective scales, resulting in subjective scales being more popular in clinical practice (5-6). At present, it is believed that objective and subjective scales evaluate different parameters in the process of improvement following nasal surgery (3). 

In our study, 84.7% of patients reported improvement in nasal obstruction following surgery. This figure was slightly higher than those in other studies; for example, in a study by Pedersen et al. (11), 76.3% of patients reported such improvement. Those authors concluded that smaller hospitals, like ours, achieve better outcomes, primarily due to the lesser complexity of the cases that they handle. We also verified that surgical treatment yielded an improvement on the NOSE scale of 42.6±22.90 points (p <0.01). A literature review revealed that significant improvements on the NOSE scale are seen following surgery, although the magnitude of improvement varies by author (Table 2) (12-17). 

**Table 2:** Pre- and post-surgical NOSE values described in the literature

	**Age median**	**NOSE pre-surgical**	**NOSE post-surgical**	**Points of improvement**	**Assessment (months)**	**N**
LaraSanchez et al^12^	44,8	53,0	30,0	23,0	2	102
Bezerra et al^13^	37,5	75,0	10,0	65,0	3	45
Shukla et al^14^	29.9	73,3	17,6	55,7	3	80
Larrosa et al^ 15^	43,9	63,4	14,0	49,4	3	58
Kahveci et al^16^	34,9	60,2	11,2	49,0	6	27
Mondina et al^17^	43,4	57,9	22,7	35,2	6	100
Our study	37,1	70,4	27,8	42,6	33	98

Lara-Sánchez et al., (12) for their part, reported an improvement with septoturbinoplasty on the NOSE scale, but by just 15 points. We think this might have been due to the fact that their preoperative NOSE scores (45±4) were quite a bit lower than ours (70.46±12.20) and, therefore, their patients might have had smaller margins for improvement. It should be kept in mind that the average NOSE scale score is 42±27 in the general population, whereas it is 65±22 in the population with nasal obstruction (10). At the other extreme, Bezerra et al. achieved a 65-point improvement on the NOSE scale (13). We believe that the sample size, which was substantially smaller than ours (45 patients), and the short follow-up period (three months) might have influenced their results. Following on from this last point, another significant consideration that could broadly alter subjective measurements is the timing of the determination of postoperative outcomes. In our series, outcomes were determined an average of 33 months after surgery, and 28.6% of those who experienced improvement found that it was only temporary with a mean duration of improvement of 4.24 months. The impact of the passage of time on the evaluation of improvement has not been taken into account in prior studies. We found no studies that measured outcomes six months after surgery. Lara-Sánchez et al., (12) Bezerra et al., Shukla et al. and Larrosa et al. conducted their evaluations in the first three months of the postoperative period; this might have rendered their results overestimates (13-15). Only Kahveci et al. and Mondina et al. conducted their evaluations at six months; their findings with regard to improvement were similar to ours (35 points and 49 points, respectively) (16,17). According to most authors, the most commonly used scale is the NOSE scale as it is validated and specific to nasal obstruction (10,12-17). In addition to being useful for evaluating nasal obstruction, it helps patients to better understand their symptoms (14).

However, we believe that, in clinical practice, the use of the VAS should be considered because it has a high index of correlation with the NOSE scale (both in our study [r = 0.79; p <0.01) and in the rest of the literature (10)) and can be used more quickly and easily than the NOSE scale (12,14). The way in which the VAS is used has varied from study to study. Authors of prospective studies tend to use the VAS to measure patients' preoperative and postoperative sensation of nasal obstruction using an incremental scale (where 0 represents no obstruction at all and 100 represents complete obstruction). One systematic review calculated VAS-measured improvement in nasal obstruction with surgical treatment at 46 points (10). As ours was a retrospective study; we found it more useful to use the VAS to measure improvement experienced in nasal obstruction following surgery, with 0 representing no improvement at all and 100 representing complete improvement. Our patients achieved an improvement following surgery of 69.80±26.97 points.

The predominant sex, both in our study (88.8%) and in the rest of the literature, was male (10,12-17). Bezerra et al. (13), with 66.1% men, did not find differences between the sexes in perceived improvement on the NOSE scale; however, in our study, women showed a higher percentage of temporary improvement (p <0.01). We believe that women's lower baseline nasal airflow could be a factor (18). It also must be remembered that women tend to have worse perceived quality of life scale scores (19). 

Most authors have found no significant differences in scores obtained on subjective scales by patient age. In our sample, patients under 30 years of age had higher rates of improvement in nasal obstruction on both the VAS (p <0.01) and the NOSE scale. We are not aware of any reason for this. Cetiner et al. warned that patients over 40 years of age may show worse functional outcomes in nasal surgery due to decreased microvascularisation of the mucosa (20). On the other hand, Pedersen et al. found the opposite effect (11), which they attributed to older patients having larger baseline nasal cavities (measured by acoustic rhinometry) and therefore more capacity for improvement in nasal airflow with surgery. 

Our department is coming to systematically add turbinoplasty to septoplasty. Dinesh et al. confirmed (21), using the NOSE scale, that patient satisfaction is higher when the two techniques are combined. According to Lara-Sánchez et al. (12), surgical treatments yield greater improvements on the NOSE scale than medical treatments, but there are no significant differences by surgical technique used (septoplasty, septoturbinoplasty or turbinoplasty alone). Mondina et al. (17) measured outcomes of septoplasty alone without turbinoplasty and found a 35-point improvement on the NOSE scale.

We found no significant differences in VAS or NOSE scale scores when we compared the scores corresponding to each of our seven surgeons. We understand that one possible explanation could be our systematic practice of having a second surgeon act as an assistant in all surgical procedures, with that assistant, at certain times, offering a second opinion during surgery. In addition, our hospital has no residents who are learning the specialisation. Although studies similar to this one have not added this variable, it is well known that surgeon experience is key to the success of this surgical technique (9). 

Another important aspect of our study was that smoking did not impact surgical outcomes when measuring with both scales (VAS p = 0.49; NOSE p = 0.64). It is known that tobacco causes a decrease in mucociliary clearance and that this is a significant factor in the sensation of nasal obstruction (22). Another factor that influences mucociliary clearance is turbulent flow caused by septal deviation (9). In view of our results, we could assume that correction of septal deviation enabled improvement in mucociliary clearance, masking the toxic effect of tobacco on nasal airflow.

 The number of postoperative complications found was lower than those reported in the literature (10,23). We think that this might have been the reason why differences in perceived surgical outcomes were not seen between patients who experienced complications and patients who did not (VAS p = 0.12; NOSE p = 0.34). Dąbrowska-Bień et al.^23^ reported a rate of adhesions of 8%-13%, a rate of haemorrhages of 6.8%-10.7% and a rate of septal perforations of 1.6%-6.7% in a study with more than 5,000 patients. In our study, these rates were 2.4%, 3.1% and 0%, respectively. 

The main limitations of our study were that it was a single-centre observational study and that preoperative NOSE scale scores were determined after patients had undergone surgery. However, we found that the outcomes seen in our study were comparable to those shown in studies by other authors who determined those scores prior to surgery, so we propose a study to verify the correlation between them.

## Conclusions

After undergoing surgical treatment for nasal obstruction due to septal dysmorphia and turbinate hypertrophy, patients experienced a significant improvement in NOSE scale scores. The improvement experienced in nasal obstruction measured by the VAS was 69.80±26.97 points.

Improvements in nasal obstruction measured with the VAS and the NOSE scale showed a strong, direct correlation.

We found no significant differences in terms of VAS and NOSE scale results depending on the surgeon who performed the procedure.

Women showed a stronger tendency to perceive their improvement as temporary.

Better VAS and NOSE scale scores were associated with age under 30 years.
